# Ce^3+^ and Tb^3+^ doped Ca_3_Gd(AlO)_3_(BO_3_)_4_ phosphors: synthesis, tunable photoluminescence, thermal stability, and potential application in white LEDs

**DOI:** 10.1039/c8ra01322e

**Published:** 2018-03-09

**Authors:** Bin Li, Qi Sun, Shaoying Wang, Heng Guo, Xiaoyong Huang

**Affiliations:** Key Lab of Advanced Transducers and Intelligent Control System, Ministry of Education and Shanxi Province, College of Physics and Optoelectronics, Taiyuan University of Technology Taiyuan 030024 PR China huangxy04@126.com

## Abstract

Novel blue-green-emitting Ca_3_Gd(AlO)_3_(BO_3_)_4_:Ce^3+^,Tb^3+^ phosphors were successfully synthesized *via* traditional high temperature solid reaction method. X-ray diffraction, luminescence spectroscopy, fluorescence decay time and fluorescent thermal stability tests have been used to characterize the as-prepared samples. The energy transfer from Ce^3+^ to Tb^3+^ ions in the Ca_3_Gd(AlO)_3_(BO_3_)_4_ host has been demonstrated to be by dipole–dipole interaction, and the energy transfer efficiency reached as high as 83.6% for Ca_3_Gd_0.39_(AlO)_3_(BO_3_)_4_:0.01Ce^3+^,0.6Tb^3+^. The critical distance was calculated to be 9.44 Å according to the concentration quenching method. The emission colour of the obtained phosphors can be tuned appropriately from deep blue (0.169, 0.067) to green (0.347, 0.494) through increasing the doping concentrations of Tb^3+^. Moreover, the Ca_3_Gd_0.39_(AlO)_3_(BO_3_)_4_:0.01Ce^3+^,0.6Tb^3+^ phosphor possessed excellent thermal stability at high temperature, and the emission intensity at 423 K was about 87% of that at 303 K. Finally, the fabricated prototype LED device with a BaMgAl_10_O_7_:Eu^2+^ blue phosphor, CaAlSiN_3_:Eu^2+^ red phosphor, Ca_3_Gd_0.39_(AlO)_3_(BO_3_)_4_:0.01Ce^3+^,0.6Tb^3+^ green phosphor and 365 nm-emitting InGaN chip exhibited bright warm white light. The current study shows that Ca_3_Gd_0.39_(AlO)_3_(BO_3_)_4_:0.01Ce^3+^,0.6Tb^3+^ can be used as a potential green phosphor for white LEDs.

## Abstract 2

Novel thermal-stable blue-green-emitting Ca_3_Gd(AlO)_3_(BO_3_)_4_:Ce^3+^,Tb^3+^ phosphors were developed for near-ultraviolet-excited white LEDs.

## Graphical abstract

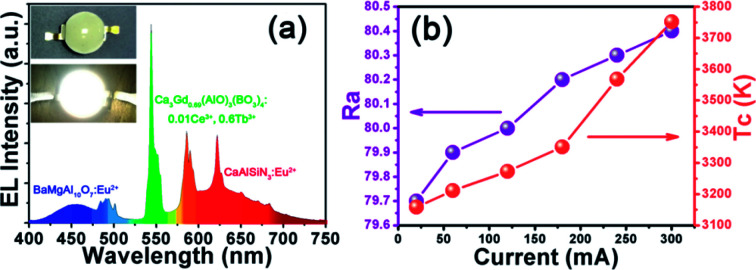

## 1. Introduction

During the past decades, inorganic luminescent materials based on rare-earth (RE) ions have attracted many researchers' attention because of their wide applications, such as lighting, displays, lasers, solar cells, sensors and bioimaging.^1–19^ For lighting, the research on phosphor-converted white light-emitting diodes (pc-WLEDs) is prevalent because they possess many merits, including low electricity consumption, long service life, small volume, non-pollution, and fast response.^20^ RE ions can emit various different fascinating colours due to their special 4f shell configuration. Through combining different RE ions and controlling their respective concentration, the emission of phosphor can be tuned from violet to red.^21–37^ Therefore, the use of different RE ions can produce a variety of colour tunable phosphors.^38–40^ As one important part of RGB (red, green and blue) phosphors for ultraviolet (UV)-pumped WLEDs, green phosphors are always fabricated by Tb^3+^ ion as its intrinsic green emissions peaking at around 544 nm.^41^ However, Tb^3+^ activated phosphors are inefficient because their absorption lines of 4f–4f spin-forbidden transitions are too weak. As a result, researchers introduce Ce^3+^ ions as a sensitizer to intensify Tb^3+^ ions absorption. Furthermore, the broad f–d absorption band of Ce^3+^ ions locate on UV region, which maximally broadens the application of Tb^3+^ ions.^42^ But to successfully realize that, not only request Ce^3+^ and Tb^3+^ ions but also need a proper host that can influence RE ions luminescent properties.

Borates have been proved to be outstanding host materials for inorganic phosphors, because they have many advantages, including high chemical stability, good thermal stability, and low synthetic temperature.^43,44^ Here in this work, we reported the luminescent properties of novel Ce^3+^ and Tb^3+^ co-activated Ca_3_Gd(AlO)_3_(BO_3_)_4_ phosphors. The crystal structure and the site substitution were investigated to verify the purity of phosphors and clarify the effect of doping on crystal structures. The luminescent performance including excitation, emission spectra, decay time, and chromaticity diagram was discussed to elucidate the luminescent mechanism of doping ions and the energy transfer mechanism between Ce^3+^ and Tb^3+^ in the Ca_3_Gd(AlO)_3_(BO_3_)_4_ host. Moreover, we examined their thermal stability and LED device performance for the practical application.

## 2. Experimental

A series of Ca_3_Gd_(0.99−*x*)_(AlO)_3_(BO_3_)_4_:0.01Ce^3+^,*x*Tb^3+^ (*x* = 0, 0.02, 0.05, 0.1, 0.2, 0.3, 0.4, 0.5 and 0.6) samples were successfully fabricated *via* a conventional high-temperature solid-state reaction technique. H_3_BO_3_ (analytical reagent), CaCO_3_ (analytical reagent), Gd_2_O_3_ (99.99%), Al_2_O_3_ (analytical reagent), Ce(NO_3_)_3_·6H_2_O (99.99%) and Tb(NO_3_)_3_·6H_2_O (99.99%) were used as raw materials. According to the stoichiometric ratio, these raw materials were weighted and ground in an agate mortar to achieve uniformity. In order to compensate the volatilization, the amount of H_3_BO_3_ is in excess of 5 wt%. Then, these uniform mixtures were put in the alumina crucibles and sintered at 1100 °C for 4 h in CO atmosphere to reduce the Ce ion into tri-valence ion. After that, the furnace was cooled down naturally to room temperature, and the final products were ground and collected for further characterization.

The X-ray diffraction (XRD) patterns of the samples were recorded on a Bruker D8 X-ray diffractometer using Cu Kα radiation ranging with 5–80° at step rate of 0.02°. The morphology properties of the samples were obtained by a field-emission scanning electron microscope (FE-SEM; MAIA3 TESCAN). The room-temperature photoluminescence (PL) and PL excitation (PLE) spectra and luminescence decay lifetimes of phosphors were measured by Edinburgh FS5 spectrometer equipped with a 150 W continued-wavelength Xenon lamp and a pulsed Xenon lamp, respectively. Temperature-dependent PL spectra were recorded by using the same spectrophotometer and detectors equipped with a temperature controller. The internal quantum efficiency (IQE) of the phosphors was measured on an Edinburgh FS5 spectrometer equipped with an integrating sphere coated with BaSO_4_.

The commercial blue phosphor BaMgAl_10_O_7_:Eu^2+^, red phosphor CaAlSiN_3_:Eu^2+^ and our green phosphor Ca_3_Gd_0.39_(AlO)_3_(BO_3_)_4_:0.01Ce^3+^,0.6Tb^3+^ were mixed with silicone thoroughly, and the obtained phosphor–silicone mixture was coated on the surface of the LED chips to fabricate WLED device. The photoelectric properties of the fabricated devices were measured by using an integrating sphere spectroradiometer system (HAAS-2000, Everfine). The LEDs were operated at 3 V with various driven currents of 20, 60, 120, 180, 240 and 300 mA, respectively. The spectral power distributions of LEDs were measured using a corrected spectrometer to calculate their values of correlated colour temperature (CCT) and colour rendering index (CRI).

## 3. Results and discussion

### 3.1 Phase and structure

**Fig. 1:**
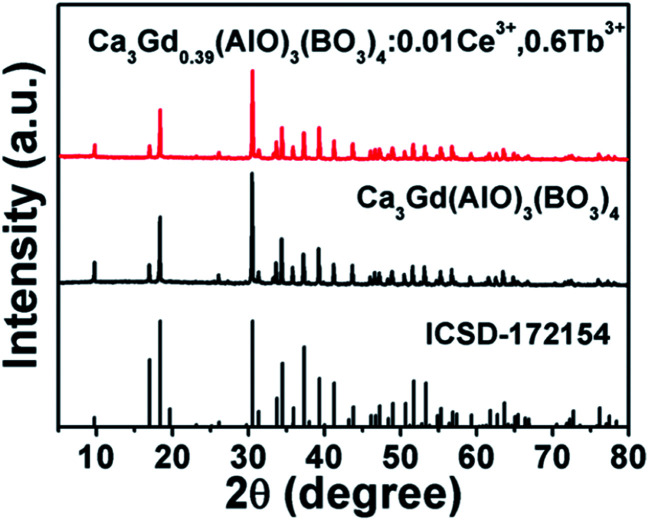
XRD patterns of Ca_3_Gd_0.39_(AlO)_3_(BO_3_)_4_:0.01Ce^3+^,0.6Tb^3+^ and Ca_3_Gd(AlO)_3_(BO_3_)_4_ phosphors. The standard data of CYAB (ICSD-172154) was shown.

Fig. 1 shows the XRD patterns of Ca_3_Gd(AlO)_3_(BO_3_)_4_ and Ca_3_Gd_0.39_(AlO)_3_(BO_3_)_4_:0.01Ce^3+^,0.6Tb^3+^ phosphors. Obviously, the obtained patterns agreed well with Ca_3_Y(AlO)_3_(BO_3_)_4_, which can be found in Inorganic Crystal Structure Database (ICSD-172154), suggesting that the compounds were obtained as a single phase.

**Fig. 2:**
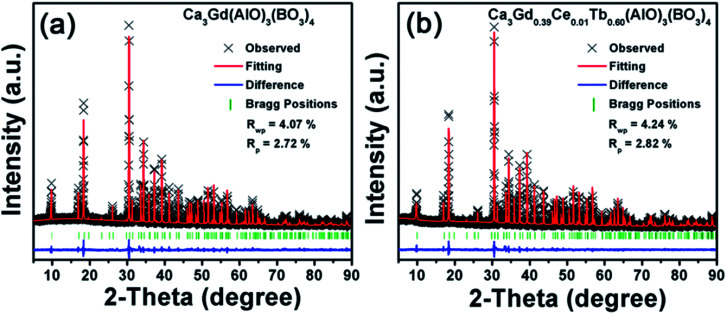
Rietveld refinements for Ca_3_Gd(AlO)_3_(BO_3_)_4_ and Ca_3_Gd_0.39_Ce_0.01_Tb_0.6_(AlO)_3_(BO_3_)_4_.

Based on the *iso*-structure of Ca_3_Y(AlO)_3_(BO_3_)_4_, the Rietveld refinements of the Ca_3_Gd(AlO)_3_(BO_3_)_4_ and Ca_3_Gd_0.39_(AlO)_3_(BO_3_)_4_:0.01Ce^3+^,0.6Tb^3+^ were performed to further analyze the crystal structure details, as shown in Fig. 2. It can be found that Ca_3_Gd(AlO)_3_(BO_3_)_4_ crystallized in a hexagonal unit cell with space group *P*63/*m*, which coincide with Ca_3_Y(AlO)_3_(BO_3_)_4_. Moreover, crystallographic data and details were summarized in Table 1, and the refined lattice parameters are changed slightly through substituting Y^3+^(1.075 Å) ions with Gd^3+^(1.107 Å), Ce^3+^(1.196 Å) and Tb^3+^(1.095 Å) ions. It can be attributed to the different radii of these ions.^45^

**Table 1:** Rietveld refinement results and lattice parameters for Ca_3_Gd(GaO)_3_(BO_3_)_4_ and Ca_3_Gd_0.39_Ce_0.01_Tb_0.6_(AlO)_3_(BO_3_)_4_ from the GSAS Rietveld refinement

Formula	Ca_3_Y(AlO)_3_(BO_3_)_4_	Ca_3_Gd(AlO)_3_(BO_3_)_4_	Ca_3_Gd_0.39_Ce_0.01_Tb_0.6_(AlO)_3_(BO_3_)_4_
Crystal system	Hexagonal	Hexagonal	Hexagonal
Space group	*P*63/m	*P*63/m	*P*63/m
2*θ*-interval,°	10–90	10–90	10–90
*a* (Å)	10.3877	10.4270	10.4244
*b* (Å)	10.3877	10.4270	10.4244
*c* (Å)	5.6920	5.7041	5.7035
*V* (Å^3^)	531.91	537.07	536.75
*R*_wp_ (%)	—	4.07%	4.24%
*R*_p_ (%)	—	2.72%	2.82%

**Fig. 3:**
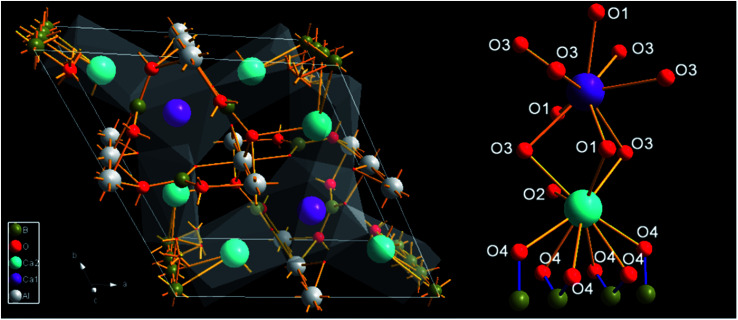
The crystal of Ca_3_Gd(AlO)_3_(BO_3_)_4_ and local environment at Ca1 and Ca2 sites.

The crystal structure of Ca_3_Gd(AlO)_3_(BO_3_)_4_ and coordination (CN) environments of Ca^2+^ sites (Ca1 and Ca2) are depicted in Fig. 3. Clearly, Ca2 sites are surrounded with ten oxygen atoms while Ca1 sites are coordinated with nine oxygen atoms, but the coordinative six oxygen (O4) ions of Ca2 are also shared by the adjacent four B ions. Therefore, the CN sites of Ca1 and Ca2 are 9 and 7, respectively. The above results suggest that Ln^3+^ (Gd^3+^, Ce^3+^ and Tb^3+^) ions prefer to occupy the Ca1 sites.^46^

**Fig. 4:**
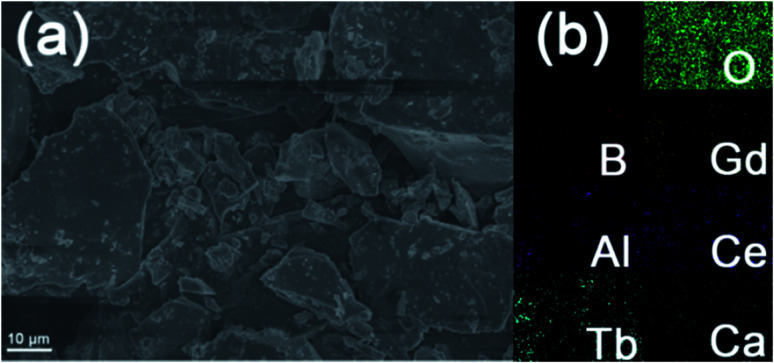
(a) The SEM and (b) elements mapping of Ca_3_Gd_0.39_(AlO)_3_(BO_3_)_4_:0.01Ce^3+^,0.6Tb^3+^.

Fig. 4(a) shows the representative FE-SEM image of Ca_3_Gd_0.39_(AlO)_3_(BO_3_)_4_:0.01Ce^3+^,0.6Tb^3+^ phosphors. As can be seen, the studied sample was made up of irregular and aggregated microparticles with the size ranging from 2 to 10 μm. Besides, the elemental mapping result revealed that the components of Ca, Ga, Al, O, B, Ce and Tb were uniformly distributed over the whole range of particles, as can be seen in Fig. 4(b).

### 3.2 Photoluminescence properties

**Fig. 5:**
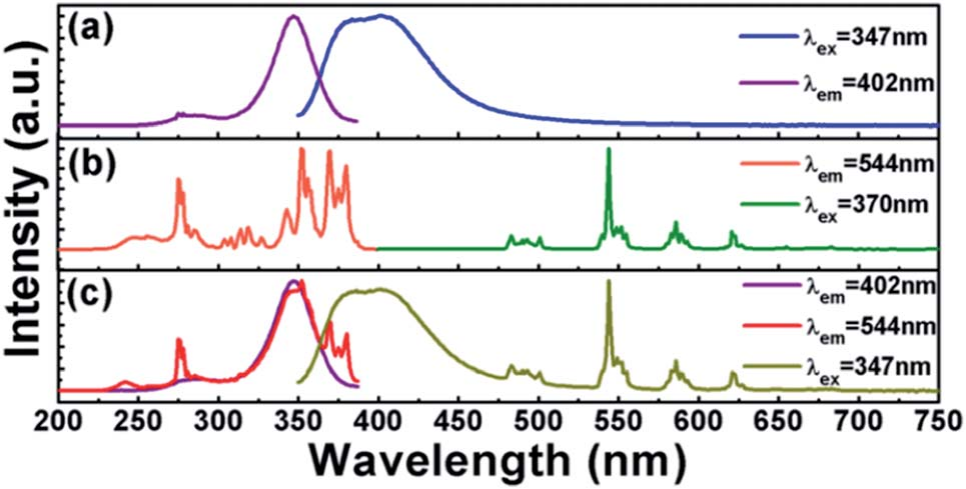
PLE and PL spectra of (a) Ca_3_Gd_0.99_(AlO)_3_(BO_3_)_4_:0.01Ce^3+^, (b) Ca_3_Gd_0.95_(AlO)_3_(BO_3_)_4_:0.05Tb^3+^ and (c) Ca_3_Gd_0.94_(AlO)_3_(BO_3_)_4_:0.01Ce^3+^,0.05Tb^3+^.

Fig. 5(a) shows the PLE and PL spectra of Ca_3_Gd_0.99_(AlO)_3_(BO_3_)_4_:0.01Ce^3+^ phosphor. By monitoring at 402 nm, there were two principle excitation bands in the PLE spectrum: the first one was located in the 250–300 nm region and the second one was located in the range of 300–380 nm with a peak at around 347 nm, originating from 4f → 5d transitions of Ce^3+^ ions.^22^ The sharp PLE peak at 276 nm was attributed to the ^8^S_7/2_→^6^I_7/2_ transition of Gd^3+^ ions, indicating the energy transfer from Gd^3+^ to Ce^3+^ ions in the Ca_3_Gd_0.99_(AlO)_3_(BO_3_)_4_:0.01Ce^3+^ phosphor.^47^ The PL spectrum was a broad emission band centered at 402 nm, corresponding to the transition from the 5d level to the ground state of the Ce^3+^ ion.

The PLE and PL spectra of the representative phosphor Ca_3_Gd_0.95_(AlO)_3_(BO_3_)_4_:0.05Tb^3+^ were presented in Fig. 5(b). The PLE spectrum was recorded by monitoring with bright green emission at 544 nm, which revealed a series of excitation bands in the range of 230 to 400 nm. The broad PLE band in the range of 230–300 nm was due to the 4f–5d transitions of Tb^3+^ ions. The PLE peak at 276 nm can be attributed to the ^8^S_7/2_ → ^6^I_7/2_ transition of Gd^3+^ ions, indicating the occurrence of energy transfer from Gd^3+^ to Tb^3+^ ions.^48^ In the 300–400 nm wavelength region, a series of PLE peaks were observed, which can be attributed to the 4f–4f electronic transitions of Tb^3+^ ions, such as ^7^F_6_ → ^5^H_7_ at 314 nm, ^7^F_6_ → ^5^L_7_ at 343 nm, ^7^F_6_ → ^5^D_2_ at 352 nm, ^7^F_6_ → ^5^G_6_ at 370 and ^7^F_6_ → ^5^D_3_ at 380 nm. Upon 370 nm excitation, strong green emission was presented in the emission spectrum. The emission peaks at 492, 544, 586, and 622 nm can be assigned to ^5^D_4_ → ^7^F_*J*_ (*J* = 6, 5, 4, 3) transitions, respectively.^41^

By comparison of the Fig. 5(a) and (b), we can observe the overlap between the emission band of Ce^3+^ and the f–f excitation of Tb^3+^, indicating the possible resonance energy transfer from Ce^3+^ to Tb^3+^ in Ca_3_Gd(AlO)_3_(BO_3_)_4_ host. For further identifying it, we measured the PLE and PL spectra of Ce^3+^ and Tb^3+^ co-doped sample Ca_3_Gd_0.85_(AlO)_3_(BO_3_)_4_:0.01Ce^3+^,0.05Tb^3+^, as shown in Fig. 5(c). Clearly, not only the characteristic emission bands of Tb^3+^ can be observed under excitation at 347 nm, but also the two broad PLE bands of Ce^3+^ also existed when monitored at 544 nm. The results above show the energy transfer from Ce^3+^ to Tb^3+^ truly happened in Ca_3_Gd(AlO)_3_(BO_3_)_4_ host.

**Fig. 6:**
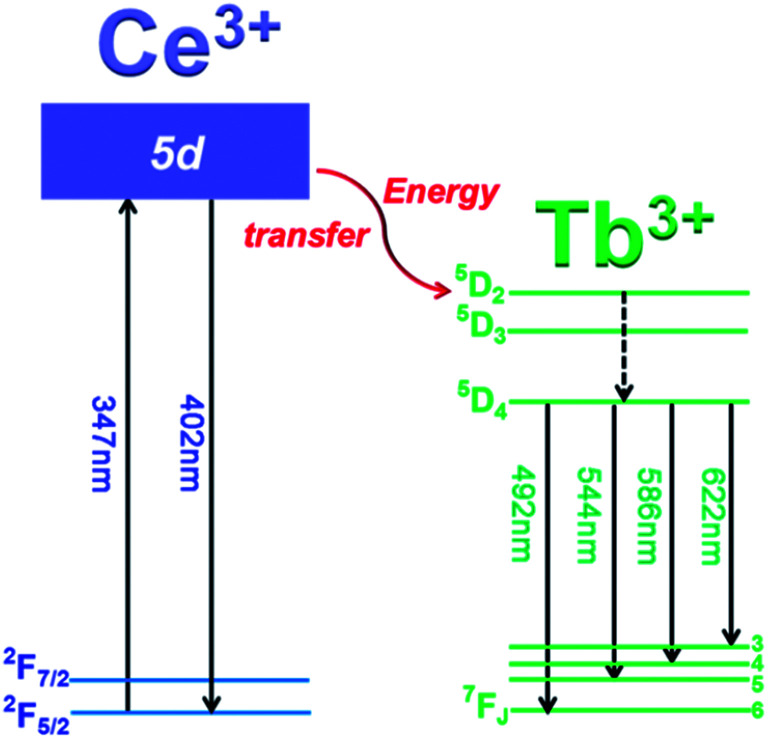
Illustration of energy levels of Ce^3+^ and Tb^3+^, and the energy transfer mechanism of Ce^3+^ → Tb^3+^.

In order to discuss the energy transfer mechanism of Ce^3+^ → Tb^3+^, the illustration of electronic transitions of Ce^3+^ and Tb^3+^ is depicted in Fig. 6. Under 347 nm excitation, the electrons on Ce^3+^ ions are excited from ground state (^2^F_7/2_) to higher excited state (5d), then some excited electrons return to ground states (^2^F_5/2_ and ^2^F_7/2_), generating blue emission of Ce^3+^. Meanwhile other excited electrons transfer part of their energy from Ce^3+^ 5d level to the excited levels of Tb^3+^ (^5^D_2_) through cross-relaxation, and subsequently, Tb^3+^ relaxes non-radiatively to the ^5^D_4_ level from ^5^D_2_ and ^5^D_3_ states. Finally, radiative transitions take place through ^5^D_4_ → ^7^F_J_ (*J* = 3–6) transitions, giving rise to the characteristic green emissions of Tb^3+^.

**Fig. 7:**
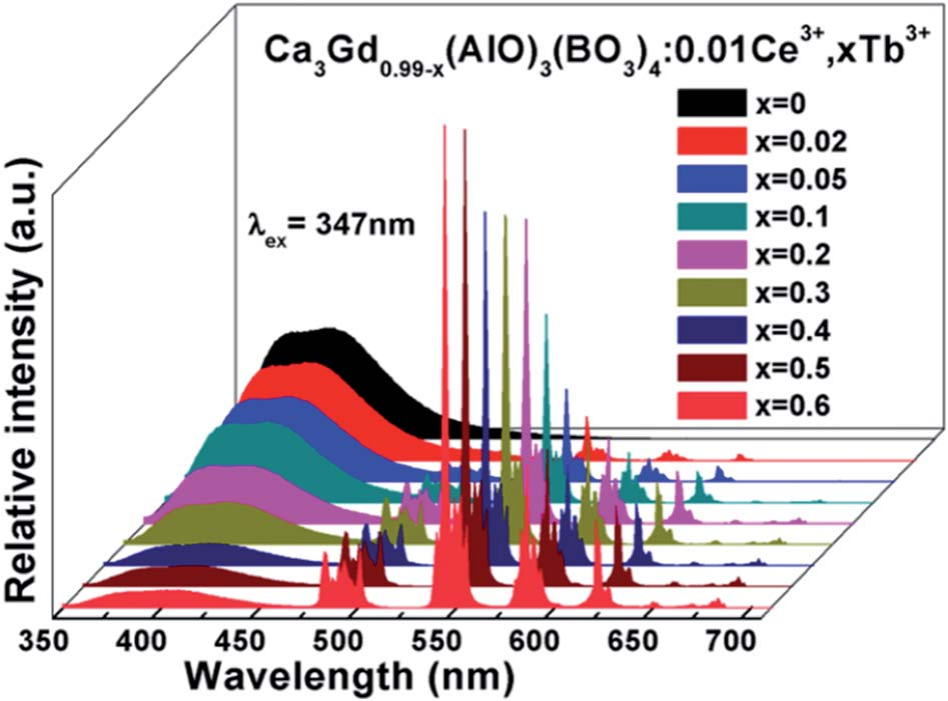
PL spectra of Ca_3_Gd_(0.99−*x*)_(AlO)_3_(BO_3_)_4_:0.01Ce^3+^,*x*Tb^3+^ (*x* = 0, 0.02, 0.05, 0.1, 0.2, 0.3, 0.4, 0.5 and 0.6) phosphors excited at 347 nm.

**Fig. 8:**
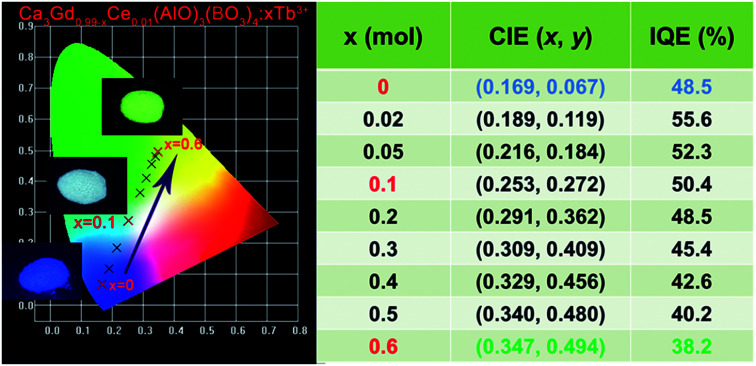
CIE chromaticity diagram showing emission colour tuning in Ca_3_Gd_(0.99−*x*)_(AlO)_3_(BO_3_)_4_:0.01Ce^3+^,*x*Tb^3+^ phosphors under single 347 nm UV excitation. Insets are photographs of the representative phosphors upon excitation under a 365 nm UV lamp.

Fig. 7 shows the PL spectra of Ca_3_Gd_(0.99−*x*)_(AlO)_3_(BO_3_)_4_:0.01Ce^3+^,*x*Tb^3+^ (*x* = 0, 0.02, 0.05, 0.1, 0.2, 0.3, 0.4, 0.5 and 0.6) phosphors under excitation at 347 nm. As can be seen, the PL spectra of Ce^3+^/Tb^3+^ co-doped Ca_3_Gd(AlO)_3_(BO_3_)_4_ samples consisted of blue emission band of Ce^3+^ ions and the characteristic green emission peaks of Tb^3+^ ions, due to the energy transfer from Ce^3+^ to Tb^3+^. Moreover, with increasing Tb^3+^ concentration from *x* = 0 to *x* = 0.6, the emission intensity of Ce^3+^ ions decreased monotonically, while the emission intensity of Tb^3+^ ions gradually increased without emission quenching. Interestingly, the emission colour of phosphors can be tuned through increasing Tb^3+^ concentration, and the corresponding Commission Internationale de L'Eclairage (CIE) chromaticity moved from blue to green, as can be seen in Fig. 8. The digital photos depict a series of the representative Ca_3_Gd_(0.99−*x*)_(AlO)_3_(BO_3_)_4_:0.01Ce^3+^,*x*Tb^3+^ (*x* = 0, 0.1, and 0.6) phosphors under 365 nm lamp. These visual images reflect the high potential of the phosphors as luminescence materials in the blue-green emission range. Besides, the IQEs of the Ca_3_Gd_(0.99−*x*)_(AlO)_3_(BO_3_)_4_:0.01Ce^3+^,*x*Tb^3+^ phosphors were also listed in Fig. 8, which can be calculated based on the formula below.^49^1
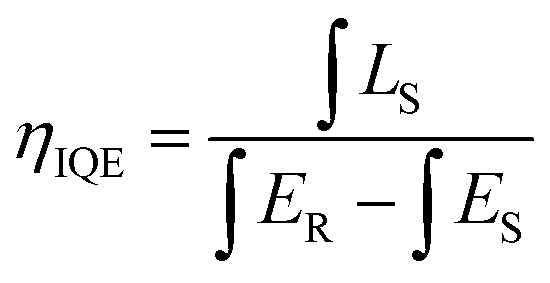
where *L*_S_ is the emission spectrum of the sample, *E*_S_ and *E*_R_ represent the excitation light with and without the sample in the integrating sphere, respectively. Herein, the Ca_3_Gd_0.97_(AlO)_3_(BO_3_)_4_:0.01Ce^3+^,0.02Tb^3+^ had the maximum value of 55.6%, and the IQE gradually decreased to be 38.2% with the increasing Tb^3+^ concentration *x* in Ca_3_Gd_(0.99−*x*)_(AlO)_3_(BO_3_)_4_:0.01Ce^3+^,*x*Tb^3+^.

### 3.3 Energy transfer mechanism

**Fig. 9:**
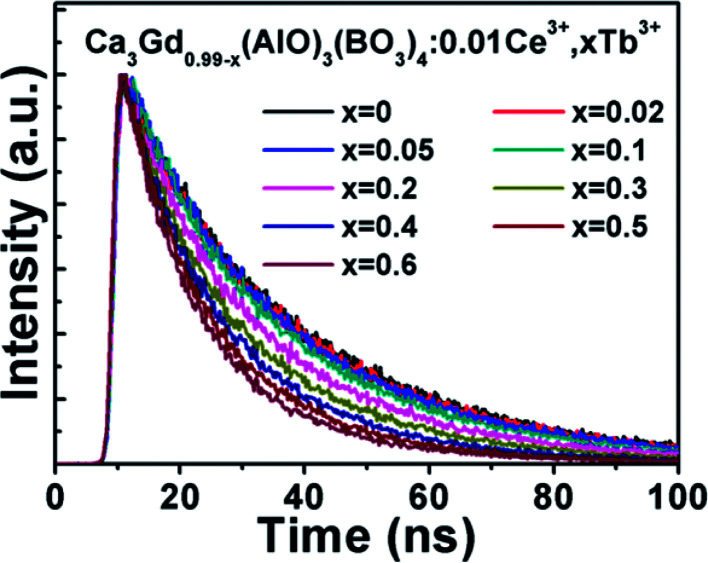
Fluorescence decay curves of Ce^3+^ in Ca_3_Gd_(0.99−*x*)_(AlO)_3_(BO_3_)_4_:0.01Ce^3+^,*x*Tb^3+^ after pulse excitation at 347 nm while monitored at 402 nm.

**Fig. 10:**
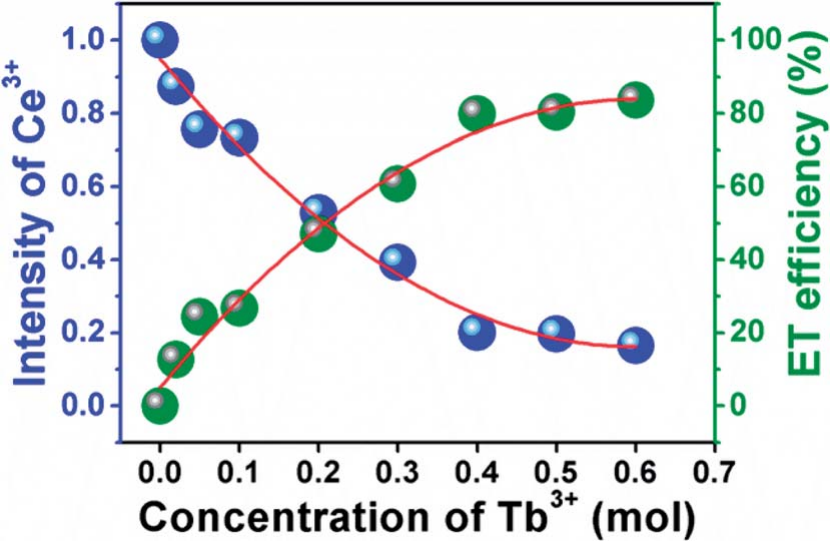
Dependence of Ce^3+^ emission intensities and energy transfer efficiency on Tb^3+^ concentration in Ca_3_Gd_(0.99−*x*)_(AlO)_3_(BO_3_)_4_:0.01Ce^3+^,*x*Tb^3+^.

The decay curves of Ca_3_Gd_(0.99−*x*)_(AlO)_3_(BO_3_)_4_:0.01Ce^3+^,*x*Tb^3+^ samples were measured for identifying the energy transfer mechanism, as shown in Fig. 9. The fluorescence average lifetimes *τ* can be obtained from following formula:2
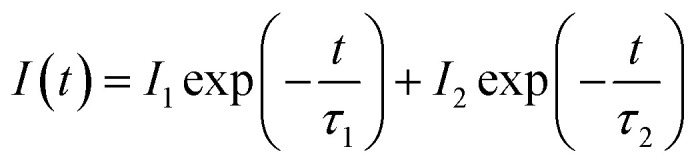
where *I*_1_ and *I*_2_ refer to intensities at different time, and *τ*_1_ and *τ*_2_ represent the corresponding decay time for the exponential components, respectively. The obtained average decay lifetimes of Ce^3+^ are determined to be 29.47, 28.93, 28.00, 26.42, 24.31, 21.61, 19.03, 17.07 and 15.76 ns with increasing contents of Tb^3+^ ions from *x* = 0 to *x* = 0.6. Obviously, the lifetime gradually decreased with increasing Tb^3+^ concentration, confirming the above-mentioned energy transfer mechanism of Ce^3+^ → Tb^3+^. The energy transfer efficiency (*η*_T_) of Ce^3+^ → Tb^3+^ can be obtained using the equation below:^42^3
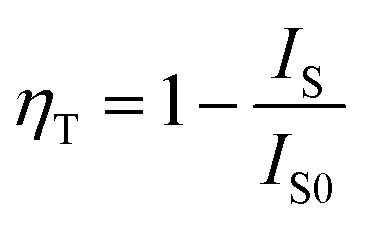
where *I*_S0_ and *I*_S_ represent the luminescence intensity of Ca_3_Gd_0.99_(AlO)_3_(BO_3_)_4_:0.01Ce^3+^ and Ca_3_Gd_(0.99−*x*)_(AlO)_3_(BO_3_)_4_:0.01Ce^3+^,*x*Tb^3+^ (*x* = 0.02, 0.05, 0.1, 0.2, 0.3, 0.4, 0.5 and 0.6). According to the dependence of the intensities of the 5d–4f transition of Ce^3+^ at 402 nm, the energy transfer efficiency of Ce^3+^ → Tb^3+^ was calculated and shown in Fig. 10. At the beginning of the process, the energy transfer efficiency raised rashly with increasing Tb^3+^ concentration. Subsequently, it grew slowly after *x* = 0.4, and eventually reached up to 83.6% when *x* = 0.6.

As it is known, the mechanism of energy transfer from Ce^3+^ to Tb^3+^ ions can be attributed to exchange interaction or electric multipolar interaction. To figure out which interaction dominated in the energy transfer process, the average distance (*R*_c_) between the Ce^3+^ donors and Tb^3+^ acceptor ions in Ca_3_Gd_0.39_(AlO)_3_(BO_3_)_4_:0.01Ce^3+^,0.6Tb^3+^ phosphor was evaluated by using the following equation:^21^4
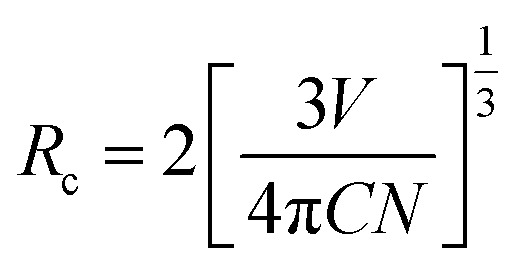
where *C* is the total concentration of Ce^3+^ and Tb^3+^ ions, *N* is coordination number, and *V* is the cell volume. For the Ca_3_Gd_0.39_(AlO)_3_(BO_3_)_4_:0.01Ce^3+^,0.6Tb^3+^ phosphor, the *V*, *C* and *N* are 536.75 Å^3^, 0.61 and 2, respectively. Accordingly, *R*_c_ was determined to be 9.44 Å. Generally, exchange interaction requires a smaller *R*_c_ value (<5 Å),^15^ and consequently the energy transfer from Ce^3+^ to Tb^3+^ in Ca_3_Gd_0.39_(AlO)_3_(BO_3_)_4_:0.01Ce^3+^,0.6Tb^3+^ phosphor would take place *via* electric multipolar interaction.

**Fig. 11:**
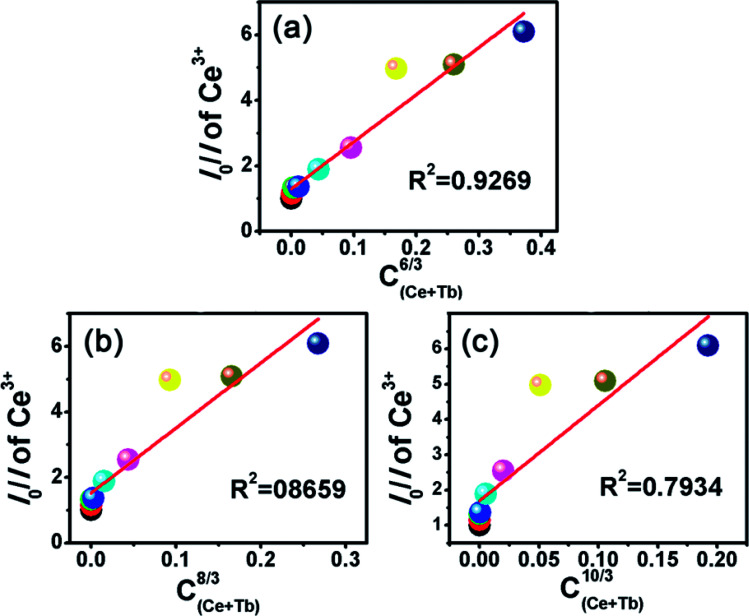
Dependence of *I*_0_/*I* on (a) *C*_(Ce+Tb)_^6/3^, (b) *C*_(Ce+Tb)_^8/3^ and (c) *C*_(Ce+Tb)_^10/3^.

As discussed by many researchers, the multipolar interaction can be further confirmed by using the following formula:^38^5
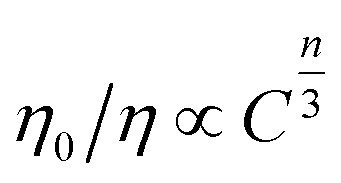
where *η*_0_ and *η* are the QEs of the Ce^3+^ in the absence and presence of Tb^3+^, respectively. The value of *η*_0_/*η* can be approximately calculated by the ratio of relative luminescence intensity ratio (*I*_S0_/*I*); *C* is the concentration of the sum of Ce^3+^ and Tb^3+^. By using eqn (5), the electric multipolar interaction parameter *n* taking the values 6 (dipole–dipole), 8 (dipole–quadrupole), and 10 (quadrupole–quadrupole) were compared by the dependence of *I*_S0_/*I* of Ce^3+^ on *C*^*n*/3^, as demonstrated in Fig. 11. A line relation was well-fitted at *n* = 6, so the energy-transfer mechanism of Ce^3+^ → Tb^3+^ could be the dipole–dipole interaction.

### 3.4 Thermal stability

**Fig. 12:**
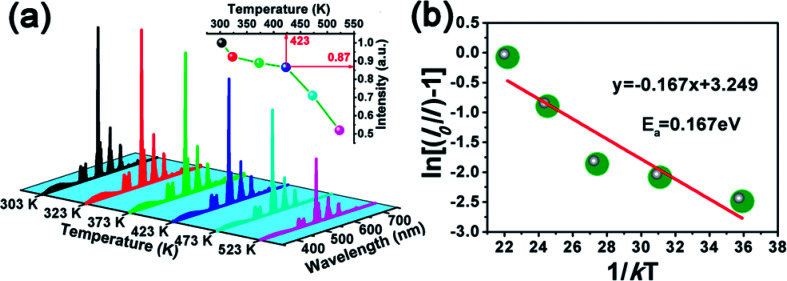
(a) Temperature-dependent PL spectra of CaGd_0.39_(AlO)_3_(BO_3_)_4_:0.01Ce^3+^,0.6Tb^3+^ phosphor excited at 347 nm. The inset shows normalized PL emission intensity of CaGd_0.39_(AlO)_3_(BO_3_)_4_:0.01Ce^3+^,0.6Tb^3+^ phosphor as a function of temperature. (b) Plot of ln(*I*_0_/*I* − 1) *versus* 1/*kT* and the calculated activation energy (*E*_a_) for the phosphor.

As a very important index, the thermal stability can influence the colour output and brightness of phosphor. Therefore, we measured the temperature-dependent emission spectra of Ca_3_Gd_0.39_(AlO)_3_(BO_3_)_4_:0.01Ce^3+^,0.6Tb^3+^ sample and the curves were shown in Fig. 12(a). Clearly, with temperature increased from 303 K to 523 K, the emission intensity of Ca_3_Gd_0.39_(AlO)_3_(BO_3_)_4_:0.01Ce^3+^,0.6Tb^3+^ decreased due to thermal quenching but still maintained the same profiles. Surprisingly, the PL intensity at 423 K was about 87% of that at 303 K, demonstrating that Ca_3_Gd_0.39_(AlO)_3_(BO_3_)_4_:0.01Ce^3+^,0.6Tb^3+^ phosphor possessed superior thermal stability and thus it suits for fabricating WLEDs.

The modified Arrhenius equation was then used to fit the thermal quenching data for activation energy calculation:^50^6
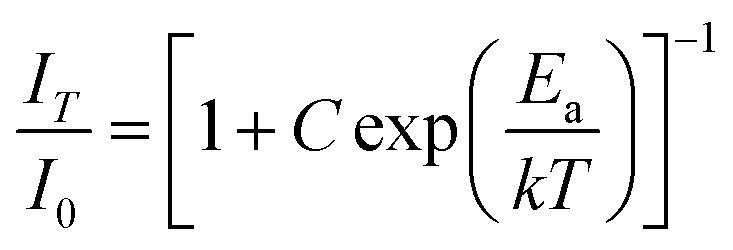
in which *I*_0_ is the initial emission intensity, *I*_*T*_ is the intensity at temperature *T*, *E*_*a*_ is the activation energy, *C* is a constant for a certain host and *k* is the Boltzmann constant, respectively. Fig. 12(b) shows the plot of ln(*I*_0_/*I* − 1) *vs.* 1/*kT*, and the experimental data can be linear fitted with a slope of −0.167. Thus, the activation energy of thermal quenching was 0.167 eV.

### 3.5 Electroluminescence properties of WLEDs fabricated with Ca_3_Gd_0.39_(AlO)_3_(BO_3_)_4_:0.01Ce^3+^,0.6Tb^3+^

**Fig. 13:**
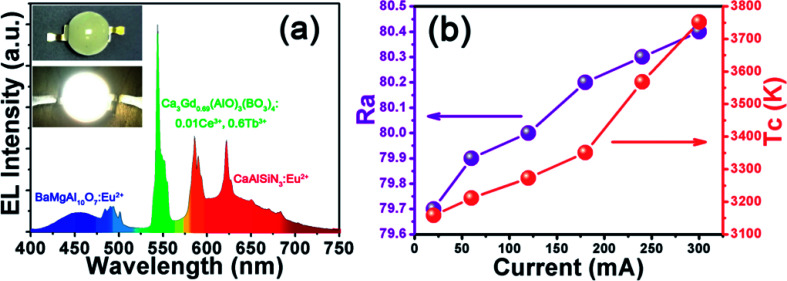
(a) EL spectrum of the fabricated WLED lamp with 365 nm NUV chip and BaMgAl_10_O_7_:Eu^2+^, CaAlSiN_3_:Eu^2+^ and CaGd_0.39_(AlO)_3_(BO_3_)_4_:0.01Ce^3+^,0.6Tb^3+^ phosphors driven by 120 mA current. (b) The variation of *R*_a_ and *T*_c_ on different driven currents.

In order to evaluate the potential application of Ca_3_Gd_0.39_(AlO)_3_(BO_3_)_4_:0.01Ce^3+^,0.6Tb^3+^ phosphor, a WLED lamp was fabricated through the combined use of a NUV chip (365 nm) and BaMgAl_10_O_7_:Eu^2+^ blue phosphor, CaAlSiN_3_:Eu^2+^ red phosphor and Ca_3_Gd_0.39_(AlO)_3_(BO_3_)_4_:0.01Ce^3+^,0.6Tb^3+^ green phosphor driven by a 20 mA current. The electroluminescence (EL) spectrum of such a WLED under the driven current of 20 mA was shown in Fig. 13(a). The bright warm white light can be seen in the inset of Fig. 13(a). The CCT, CRI (Ra), and CIE chromaticity coordinate of this WLED device was measured to be 3158 K, 79.7, and (0.427, 0.401), respectively. The above values of the WLED device under various currents were also measured (see Fig. 13(b)) and there was little variation, which confirmed the stable white light output in the device.

## 4. Conclusions

In summary, we have successfully developed a novel green emitting phosphor for near UV-pumped WLEDs. The energy transfer from Ce^3+^ to Tb^3+^ in Ca_3_Gd(AlO)_3_(BO_3_)_4_ host has been demonstrated to be the dipole–dipole interaction, and the energy transfer efficiency was as high as 83.6% on Ca_3_Gd_0.39_(AlO)_3_(BO_3_)_4_:0.01Ce^3+^,0.6Tb^3+^. The energy transfer critical distance was calculated to be 9.44 Å according to the concentration quenching method. The emission colour of the obtained phosphors can be tuned appropriately from deep blue (0.169, 0.067) to green (0.347, 0.494) by increasing the contents of Tb^3+^ ions. Besides, Ca_3_Gd_0.39_(AlO)_3_(BO_3_)_4_:0.01Ce^3+^,0.6Tb^3+^ phosphor possessed good thermal stability at high temperature, and the emission intensity at 423 K was about 87% of that at 303 K. A prototype LED device was fabricated by using the BaMgAl_10_O_7_:Eu^2+^ blue phosphor, CaAlSiN_3_:Eu^2+^ red phosphor and Ca_3_Gd_0.39_(AlO)_3_(BO_3_)_4_:0.01Ce^3+^,0.6Tb^3+^ green phosphor and 365 nm-emitting InGaN chip, and bright warm white light with CCT (3158 K), CRI (79.7), and CIE chromaticity coordinates (0.427, 0.401) was achieved. These results indicate that the Ca_3_Gd_0.39_(AlO)_3_(BO_3_)_4_:0.01Ce^3+^,0.6Tb^3+^ sample can be as a promising green-emitting phosphor for UV-based white LEDs.

## Conflicts of interest

There are no conflicts to declare.

## Supplementary Material
